# Supplementary material for: Stakeholder perspectives on an integrated package of care for lower limb disorders caused by podoconiosis, lymphatic filariasis or leprosy: A qualitative study
Source: PLoS Negl Trop Dis. 2022 Jan 21;16(1):e0010132. doi: 10.1371/journal.pntd.0010132 (PMC8809619; doi:10.1371/journal.pntd.0010132)
Supplement: Attachment — Submitted filename: Response letter FINAL.docx [file pntd.0010132.s002.docx]

Dear Editors

Re: resubmission of manuscript "Stakeholder perspectives on an integrated package of care for lower limb disorders caused by podoconiosis, lymphatic filariasis or leprosy: a qualitative study" (PNTD-D-21-01000).

Thank you for the opportunity to revise our manuscript, "Stakeholder perspectives on an integrated package of care for lower limb disorders caused by podoconiosis, lymphatic filariasis or leprosy: a qualitative study". We appreciate the reviewers’ suggestions and comments, and are grateful for the time and thought they contributed. We believe the paper is stronger because of the changes made, and here provide a detailed list of the modifications we have made.

The reviewer comments are followed by our response in italics. We attach two copies of the manuscript: one marked with the changes, and a clean version. The revision has been developed with the co-authors and the final version approved by them all.

Thank you for your consideration

Bethany Davies on behalf of the authors

# Methods

## Reviewer #1:

- The principle behind this study is sound and practical and the potential benefits undoubted.
  I had some difficulty disentangling the physical abnormalities associated with the three target diseases – podoconiosis and LF whose main pathological focus is lower limb oedema (it may be wider than this in LF) and leprosy where the main damage is neuropathic resulting in limb ulceration with surrounding inflammation in some cases leading to localised oedema. So isn’t this study focussing on the impact and resolution of lower limb disabilities rather than lymphoedema per se?

*Thank you for your comment, with which we agree – on further consultation with our colleagues we have rephrased this as lower limb disorders throughout the manuscript where the broader term is more appropriate. This also reflects the care package documents that are being used by the teams to deliver care.*

- Rather along the same lines I wonder if stigma is based on disease specific beliefs rather than syndrome specific beliefs. Certainly other studies have shown that leprosy carries very specific associations with mental and social consequences - even without limb ulceration. Does this apply to the other diseases ? If so how does this affect the questions and their analysis?

*Thank you for this comment. We agree that there can be variations between NTDs in terms of the*

*reasons why people affected by different NTDs are stigmatised, for example in regard to the causal attributions made. However, we would argue that the types of stigma, the manifestations, the impact of affected persons and their families, and the interventions that are used to reduce stigma share many characteristics; and cross-cutting/joint approaches to stigma reduction have been recommended in recent years (Hofstraat & van Brakel 2016; van Brakel et al 2019).*

- How do other diseases including NCDs fit in with this package. I realise that idiopathic or cancer related lymphoedemas are probably uncommon in this setting. But what about diabetic foot?

*Thank you for this comment, which we agree with. Within the EnDPoINT care package there was no scope to include lymphoedemas beyond those caused by podoconiosis, LF and leprosy. However, the potential to broaden integrated care for limb disorders to other diseases has been noted by the EnDPoINT team and plans for future research on this (specifically in relation to wound care) are already in place. A couple of sentences have been added on this in the ‘Strengths and Limitations’ section of the manuscript (see lines 717-720).*

## Reviewer #2

- Could better description of the MRC framework on complex interventions and the CICE framework be provided, with a particular focus on how the endpoint study and this work map to the framework. This could perhaps be provided through a figure?

*Many thanks for this comment. A detailed description (with figure) of these frameworks and how the EnDPoINT study maps on to them has been provided elsewhere in a previous journal article (Semrau et al 2020). We have now referred to this article in the manuscript (see lines 137-138).*

- The authors described that the number of participants recruited is based on a data saturation approach- yet numbers seem small for data saturation to have been achieved- particularly given the diversity of stakeholders included in the study. Could this be further clarified or amended?

*We thank the reviewer for their comment, and have amended the description of the approach in lines 153-154: “the sample size was guided by the number of key stakeholders who were identified to take part and available, as per the original study protocol[19].” We have also included text in the Limitations section, lines 708-709 “Due to the phased nature of the overall project, data collection in this study was limited by time constraints rather than primarily by data saturation.”*

- Line 150: The authors state 'all participants were encouraged to contribute and be heard- Can the authors expand on how this was done?

*Thank you – we have included the following text in line 160: “The sample size during focus groups was limited to ensure that all participants have the opportunity to speak.” And lines 169-173: “All participants were encouraged to contribute and be heard; the discussion facilitators set ground rules for behaviour to support participants to feel safe; they also actively tried to include all participants in the discussion and to make the participants feel their contribution was welcome and worthwhile. Those participants who were not highly verbal were encouraged to share their views.”*

- Ethics- merged FGDs with community members and people affected- how was stigma/confidentiality etc. managed? is it possible that affected persons felt unable to share their true experiences if those who may stigmatise were also part of the group discussion- could this be discussed further?

*We thank the reviewer for raising this point, and agree. We have expanded the limitations section from lines 711-716 with the following sentences: “… the merged nature of focus group 1, which held both affected persons and community members. While this at times stimulated discussion, there is the risk that, despite the ground rules and the facilitators, affected persons may have felt restricted from fully sharing their experiences due to the presence of those by whom they may be stigmatized. In retrospect, including affected persons within the key informant interviews could have offset this, as well as recruiting more affected persons as participants.”*

- Further, where issues of mental distress may have been disclosed by participants how was this managed and supported?

*Thank you for this comment. The following sentence has been added to the manuscript (lines 193-195): “In the event that any participant was deemed to need immediate assistance or support, for instance because they disclosed mental distress, appropriate action was established according to local resources and capacities.”*

- Line 181-184 belongs in methods otherwise reader left guessing on group composition in the methods

*We agree and have moved this to the methods section, lines 148-161 instead.*

Reviewer #3:

- Clear methods, well crafted description of rationale and background
  *Thank you*

# Results

## Reviewer #1:

- Can you break down the groups studied further for instance to include some numbers eg how many lymphoedema patients were in the stakeholder group.

*We thank the reviewer for this suggestion. We have added to the information provided in lines 159-160 as follows: “There were three affected persons (two female and one male) in the community representative groups.” We have not provided further breakdown of numbers in order to ensure we do not inadvertently identify participants.*

- Although this is explained in the supplementary data it would be useful to indicate in the text if the questions were asked were about each disease or the syndrome ie a disabled or swollen limb.

*We have included the following text in lines 168-169 to address this point as suggested: “Questions were based around participants’ experiences regarding affected persons with lower limb disorders secondary to podoconiosis, LF or leprosy.”*

- Please expand on the costs of joining a patients’ organisation. Can you give an approximate figure?

*Thank you, we agree this would be informative to include. We have added the estimated cost to line 499:*

*“Also for the individual, costs of joining the associations [10 Ethiopian Birr / US $0.22 per person] could be prohibitive”*

## Reviewer #2:

- The results match the analysis plan well, however, a little more clarity could be provided in the results to support the reader to follow clearly.
  In Table One- as well as providing a summary of theme titles, could a summary of the content of each theme also be provided- this would help anchor the reader as they move through the results text. Otherwise this is relatively long and easy to get lost.

*We thank the reviewer for highlighting the risk of losing the reader in the results section. We have expanded the detail in Table 1 to help, and we have created Figure 1 which presents a diagrammatic overview of the results as a visual support for the reader.*

- Could quotes that do not flow directly in the text also be indented. Currently, the text flows as one long narrative in places which makes it hard for the reader to differentiate between the analytical point presented in the results and the supporting evidence. Perhaps sub-sub themes could also be bolded when referred to to make this clear to the reader.

*We thank the reviewer for highlighting this and have modified with indentations and bolding as suggested.*

- Matter matters- could this theme heading be adjusted it's a bit confusing- perhaps material matters would be a better term?

*We agree, and have amended this to “material matters” in both the table and the text.*

## Reviewer #3:

- Inclusion of the free text of some of the responses whilst increasing the length of the paper provides a richness and widespread applicability which other disease groups might find informative.

*Thank you*

# Conclusions

## Reviewer #1:

- Can you indicate how this will translate into addressing the concerns raised by the various groups?

*Further details have been added in the Discussion section of the manuscript to highlight some of the concerns raised by the various participant groups.* *We have also added the following sentence to the Strengths and limitations section, lines 700-702: “*Furthermore the inclusion of implementers and policy makers alongside other stakeholders as part of the Consortium means that they have been kept abreast of research findings throughout the study and any concerns raised have been fed into later stages of the project.”

## Reviewer #2:

- The conclusions are largely supported by the data, however, some of the less positive points related to service integration could be further emphasised, particularly related to stakeholders concerns related to integration including limited availability of space to provide a separate clinic but a wish to do so to mitigate stigma; challenges with shifting leprosy from TB service integration; and overburdening of health staff.

*We thank the reviewer for these comments, and agree that emphasising the negatives is also essential. We have amended this section from line 685 as follows:*

*However, there were also some concerns mentioned by our study participants, particularly in regard to integration of care for leprosy, since: leprosy programmes have traditionally been linked with those for tuberculosis in Ethiopia; leprosy can manifest itself in ways other than lower limb lymphoedema; and misinformation about the transmissibility of leprosy possibly limits acceptability of integrated services by people with podoconiosis and LF. Disentangling leprosy care from its current place within the TB programme may have negative sequelae, such as loss of expertise, and a requirement for extra resources, unless carefully managed. These kinds of challenges and concerns need to be addressed when planning and implementing integrated programmes. Interestingly, since completion of the EnDPoINT project, the Ethiopian Federal Ministry of Health has included leprosy into the priority NTD list /group in the 3rd National NTD Master Plan.”*

*With respect to the reviewer’s comment on the lack of space as an issue – we have included text from line 639 as follows: “Reassuringly, although the desire to provide a separate clinic to mitigate stigma would require extra space, this was not seen as a barrier of note by our participants.”*

*Lines 636-9 has been amended to acknowledge the risk of adding to the workload: “Although there may be concerns that the package adds a burden of work, both integration of care across diseases and integration of holistic care (as opposed to vertical programmes) may improve the efficiency and cost-effectiveness of care, since resources – financial, material and human – are shared.*

- The limitations could also further emphasise lack of direct (non-group specific) involvement of affected persons- their inclusion in whole group discussions may have affected their responses- or some discussion of how this was mitigated should be provided.

*We thank the reviewer, and agree, with changes made to lines 710-716: “An additional consideration is the limited inclusion of affected persons as participants. This was compounded by the merged nature of focus group 1, which held both affected persons and community members. While this could stimulate discussion, there is the risk that, despite the ground rules and the facilitators, affected persons may have felt restricted from fully sharing their experiences due to the presence of those by whom they may be stigmatized. In retrospect, including affected persons within the key informant interviews could have offset this, as well as recruiting more affected persons as participants.”*

## Reviewer #3:

- The conclusions are clear and recommendations useful

*Thank you*

# Editorial and Data Presentation Modifications

## Reviewer #2: -

- Line 69- (world bank)- needs removing
  Line 119- Deribe PLS Map Model- needs reference
  Line 120 (census)- needs removing

*We thank the reviewer for spotting these errors and have corrected them all.*

- Could the difference between mental health and psychosocial care be better articulated in the abstract and introduction where this is referred to as two separate components?

*Thank you. We agree that we could have made this clearer. We have added the following sentence in the Introduction (see lines 92-96): “Recognizing that there can be overlaps between the two terms, here we take mental health care to mean services that aim to improve a person’s psychological or emotional wellbeing, for instance interventions aiming to reduce depression or anxiety; we take psychosocial care to mean interventions that explicitly take into account interrelating social and individual factors – stigma reduction interventions could be an example of this.”*

- Could the closing paragraph of the introduction include a more specific section on the purpose of this specific study rather than the overarching endpoint work?

*We agree with the reviewer, and have expanded the end of the introduction to focus on this specific study, and also to address the Reviewer’s following point. Lines 118-122 now read:*

*“This particular study aimed to explore the perspectives of a range of stakeholders to harness their views on the feasibility, acceptability and appropriateness of such integrated care. For the purposes of this study: feasibility is conceptualized as the capability for successful completion of the project; acceptability, as pre-intervention prospective treatment acceptability[13]; and appropriateness, the suitability of the project for the needs identified.”*

- Could you define how you have conceptualised appropriateness, feasibility and acceptability either within your introduction or at the beginning of your discussion?

*We thank the reviewer for raising this, and have addressed it in the introduction as in the above point.*

- It is great that affected persons were included in group discussions. However, it would be good to see more about the strengths and limitations of the approach in including them with other community members and how far the data collated is a true representation of their views. I would also urge caution with interpretation of the finding around the role of patient associations in integrated care- from your data- my understanding is that this view point comes from health stakeholders not patients themselves. Perhaps this should be framed as a finding that needs further interrogation from the perspective of patients?

*We agree with the reviewer and have amended the text (line 643 on): “interestingly patient associations were seen as less helpful. However, some caution should be applied due to the limited inclusion of affected persons in this study; further exploration from their perspective on the role of patient associations would be helpful.”*

*We have also stated more clearly the limited number of affected persons as participant, to more truly reflect the limited voice they have been given, as well as the effects of mixed group interviews. Lines 710-716 now read:*

*“An additional consideration is the limited inclusion of affected persons as participants. This was compounded by the merged nature of focus group 1, which held both affected persons and community members. While this could stimulate discussion, there is the risk that, despite the ground rules and the facilitators, affected persons may have felt restricted from fully sharing their experiences due to the presence of those by whom they may be stigmatized. In retrospect, including affected persons within the key informant interviews could have offset this, as well as recruiting more affected persons as participants.”*

- Overall, a good paper and I look forward to seeing the outputs of the rest of the Endpoint intervention.

*Thank you*

## Reviewer #3:

This is a well written and original paper which has potential for widespread applicability in the space of integrated approaches for NTDs

*Thank you*
